# Differential expression of C-Reactive protein and Serum amyloid A in different cell types in the lung tissue of chronic obstructive pulmonary disease patients

**DOI:** 10.1186/1471-2466-14-95

**Published:** 2014-05-30

**Authors:** Carmen Calero, Elena Arellano, Jose Luis Lopez-Villalobos, Verónica Sánchez-López, Nicolás Moreno-Mata, José Luis López-Campos

**Affiliations:** 1 Unidad Médico-Quirúrgica de Enfermedades Respiratorias, Hospital Virgen del Rocío, Sevilla, Spain; 2 Instituto de Biomedicina de Sevilla (IBiS), Hospital Universitario Virgen del Rocio, Universidad de Sevilla, Sevilla, Spain; 3 CIBER de Enfermedades Respiratorias (CIBERES), Instituto de Salud Carlos III, Madrid, Spain

**Keywords:** Chronic obstructive pulmonary disease, Chronic systemic inflammatory syndrome, Acute-phase reactants, C-reactive protein, Serum amyloid A

## Abstract

**Background:**

Chronic systemic inflammatory syndrome has been implicated in the pathobiology of extrapulmonary manifestations of chronic obstructive pulmonary disease (COPD). We aimed to investigate which cell types within lung tissue are responsible for expressing major acute-phase reactants in COPD patients and disease-free (“resistant”) smokers.

**Methods:**

An observational case–control study was performed to investigate three different cell types in surgical lung samples of COPD patients and resistant smokers via expression of the C-reactive protein (CRP) and serum amyloid A (SAA1, SAA2 and SAA4) genes. Epithelial cells, macrophages and fibroblasts from the lung parenchyma were separated by magnetic microbeads (CD326, CD14 and anti-fibroblast), and gene expression was evaluated by RT-PCR.

**Results:**

The sample consisted of 74 subjects, including 40 COPD patients and 34 smokers without disease. All three cell types were capable of synthesizing these biomarkers to some extent. In fibroblasts, gene expression analysis of the studied biomarkers demonstrated increased SAA2 and decreased SAA1 in patients with COPD. In epithelial cells, there was a marked increase in CRP, which was not observed in fibroblasts or macrophages. In macrophages, however, gene expression of these markers was decreased in COPD patients compared to controls.

**Conclusions:**

These results provide novel information regarding the gene expression of CRP and SAA in different cell types in the lung parenchyma. This study revealed differences in the expression of these markers according to cell type and disease status and contributes to the identification of cell types that are responsible for the secretion of these molecules.

## Background

Chronic obstructive pulmonary disease (COPD) is associated with important extrapulmonary manifestations including cardiovascular disease, weight loss, skeletal muscle dysfunction, depression and osteoporosis [1]. Although the pathobiology of these extra-pulmonary manifestations has not been fully determined, systemic inflammation has been implicated in the pathogenesis of the majority of these manifestations [2,3]. In fact, several authors have suggested that COPD is part of a chronic systemic inflammatory syndrome [4].

Patients with COPD display systemic inflammation, measured either as increased circulating cytokines, chemokines and acute-phase proteins or as abnormalities in circulating cells [5,6]. Acute-phase reactants are among the most studied biomarkers. In particular, C-reactive protein (CRP) is related to an accelerated decline in lung function and many other clinically relevant outcomes [7,8]. Serum amyloid A (SAA), the other major acute-phase reactant in humans, has also been shown to be elevated in COPD patients [9].

There has been considerable interest in identifying the nature of COPD-mediated systemic inflammation, as such knowledge may help predict clinical responses to therapy and identify new therapeutic targets. One important issue is identifying the origin of systemic inflammation, which is currently controversial [10]. This would be a major advancement, and doing so could have an important diagnostic impact by identifying therapeutic targets to prevent disease progression to other organs, thereby preventing comorbidities [11]. The current hypothesis holds that this inflammation is generated within the lung and produces an overflow (referred to in the literature as “spill-over”) of inflammatory mediators into the bloodstream [12].

Previous studies have described how lung tissue can synthesize acute-phase biomarkers in healthy tissue as well as in animal or cell models [13]. Additionally, our group has reported that, in addition to the liver, the lung can also synthesize CRP and SAA. Interestingly, de-novo synthesis of CRP and SAA is increased in patients with COPD compared to disease-free smokers (“resistant smokers”), and this synthesis is differentially expressed in whole tissue samples depending on the anatomical location, such as the bronchus or the parenchyma [14].

In the present study, we aimed to investigate the cell types within lung tissue that express these biomarkers. We hypothesized that the expression of acute-phase reactants in lung tissue would differ with respect to the cell type used and would vary between COPD patients and healthy smokers. The objectives of this study were to characterize the gene expression of CRP and SAA in key lung cell types, including epithelial cells, macrophages and human lung fibroblasts, and to compare the gene expression profiles of COPD patients and healthy smokers.

## Methods

### Subjects

We recruited consecutive patients from the surgical waiting list from February 2010 to June 2012 who were about to undergo elective pneumonectomy or lobectomy for suspected primary lung cancer. The study was approved by the institutional review board at Hospital Virgen del Rocío, and patients provided written informed consent prior to being included in the study. Each patient was identified upon his or her day of admission (i.e., the day before surgery was planned). Patients who were under 40 years of age, had a history of acute respiratory infection during the preceding 2 months, had been previously diagnosed with a neoplasm, had received radiotherapy or chemotherapy prior to the surgery or had suffered from chronic inflammatory disease were excluded from the study. Furthermore, time from the opening of the cutaneous layer to the extraction of the anatomical sample was measured, and cases in which this time was greater than 3 hours were also excluded from the study, as any potential stimulation of the studied biomarkers due to surgery could not be ruled out.

Medical records were checked to ensure that the patients had recently been tested for lung function. Patients whose pre-surgical spirometry results revealed a forced expiratory volume in the first second (FEV_1_)/forced vital capacity (FVC) ratio <0.7 were classified as COPD, and the remaining resistant smokers were used as control subjects. All patients completed a standardized questionnaire regarding their medical history, tobacco consumption and actual treatments. Comorbidities were evaluated using the Charlson index [15]. Furthermore, the TNM staging of the primary lesion [16] and information regarding the surgical procedure were also collected. During surgery, a portion of the lung parenchyma of approximately 1 cm^2^ in area and 0.5 cm thick that was grossly normal and distant from the injury that had motivated the intervention was selected. This separation was performed in the operating room, and the sample was immediately placed in sterile surgical biopsy ice for preservation. The rest of the surgical piece followed the usual guidelines according to hospital protocol and the patient’s clinical case. To exclude bronchial colonization that could bias our results, analyses of microbiological colonization were performed at two time points: first, during the bronchoscopy that was performed to establish the diagnosis and to study the extent of the primary lesion, and second, prior to extraction of the tissue by obtaining an intrabronchial sample from the resected bronchus.

### Laboratory techniques

The collected samples consisted of pulmonary parenchyma tissues from each of the patients (COPD cases and controls).To determine the gene expression levels of acute-phase reactants in different cell types, we performed cell disintegration of the fibroblasts, epithelial cells and macrophages. Disintegration consisted of mechanical grinding using a scalpel to achieve fragmentation and treatment with enzymes in a digestion medium composed of RPMI, 5% fetal bovine serum (FBS), 1% streptomycin/penicillin,1% L-glutamine (all from PAA Laboratories, Gmb, Germany), 0.1 mM 2-β-mercaptoethanol (Sigma, GmbH, Germany), 1 mg/mL collagenase I (Sigma, GmbH, Germany) and 0.02 mg/mL DNase I. The samples were maintained in this medium at 37°C for 25 minutes and were then neutralized with collagenase and DNase in RPMI supplemented with 5% FBS, 1% streptomycin/penicillin and 1% L-glutamine. Once neutralized, the samples were centrifuged at 1000 rpm for 10 minutes at 4°C. The supernatant was then removed, and the pellets were washed with a solution of PBS/10 mM EDTA. Next, the samples were filtered at 40 microns (Becton Dickinson, NJ, USA) and were again centrifuged at 1000 rpm for 5 min at 4°C, followed by removal of the supernatant. We performed erythrocyte lysis on the pellets (Erythrocyte lysis buffer, QIAGEN, Gmb, Germany) following the manufacturer’s protocol. Separation of the different cell types was performed by binding cell-specific antibodies to magnetic beads (MACS microbeads, Miltenyi Biotec, Bergisch Gladbach, Germany) according to the manufacturer’s instructions. The selected markers were CD326 (for epithelial cells), CD14 (for macrophages) and anti-fibroblast (for fibroblasts). After incubating the samples with the various antibodies, they were passed through a magnetic separation column (MACS MS column, Miltenyi Biotec, Bergisch Gladbach, Germany). Then, the cells remaining in the columns were collected (CD326+, CD14+ or fibroblasts), and RNA extraction was performed using the TRIzol-chloroform method (TRIsureTM, Bioline Ltd., United Kingdom). The RNA was treated with RNase-free DNase using a commercial kit (QIAgen, GmbH) to remove any residual genomic DNA, and cDNA was synthesized using an iScript kit (Bio-Rad, CA). Each reaction was duplicated at a total volume of 25 μL and contained 2 μL cDNA (40 ng/μL) and 12.5 μL SYBR Green. Cell purity was determined by flow cytometry in all of the samples. The results indicated cell purity values close to 90%, with no major differences among samples.

PCR master mix (Stratagene, CA) and 10.5 μL primers/H2O were then used. RT-qPCR was performed using a MX3005P system (Stratagene) at 95°C for 30 s, 60°C for 1 min and 72°C for 30 s. Gene amplification was normalized to 18 s RNA expression. The primers used for amplification are described in Table 1. Because human SAA protein consists of 3 tightly linked genes (SAA1, SAA2 and SAA4) [17], the GenBank database from the National Center for Biotechnology Information (NCBI) was consulted, and portions of the genes that were not in the homology region were selected. These primers were then synthesized ad hoc by an external company (Sigma-Aldrich). Gene expression analysis was performed following the 2^-ΔCt^ method [18].

**Table 1:** Primers used in this study

	**Forward**	**Reverse**
18 s	5*'*-TGAAATATCCAGAACATCTTA-3*'*	5*'*-GCAAAATTTATTGTCCCATCAT-3*′*
CRP	5*'*-GTGTTTCCCAAAGAGTCGGATA-3*'*	5*'*-CCACGGGTCGAGGACAGTT-3*'*
SAA1	5*'*-ATCAGCGATGCCAGAGAGAAT-3*'*	5*'*-GTGATTGGGGTCTTTGCCA-3*'*
SAA2	5*'*-AGCCAATTACATCGGCTCAG-3*'*	5*'*-ATTTATTGGCAGCCTGATCG-3*'*
SAA4	5*'*-GTCCAACGAGAAAGCTGAGG-3*'*	5*'*-AGTGACCCTGTGTCCCTGTC-3*'*

### Statistical analysis

Statistical analysis was performed using the Statistical Package for Social Sciences (SPSS), version 20.0 (IBM Corporation, Somers, NY, USA). Absolute and relative frequencies were used to describe qualitative variables. Quantitative variables were expressed in terms of means and standard deviations. The analysis of RFA expression at the cellular level between COPD patients and resistant smokers was performed using the Mann–Whitney *U* test. Correlations were assessed using Spearman’s coefficient. Logistic regression analysis was used to investigate whether significant differences between the two study groups persisted after adjustment for body mass index, age, gender, FEV_1_ and the Charlson index. The accepted alpha error was 0.05.

## Results

### Patients and procedures

The sample consisted of 74 subjects, including 40 COPD patients and 34 disease-free smokers. The characteristics of these patients are summarized in Table 2. The distribution of patients according to their spirometric classification per GOLD 2014 was as follows: 15 (37.5%) with mild impairment (FEV_1_ = 88.3, 8.7%), 22 (55.0%) with moderate impairment (FEV_1_ = 66.5, 7.6%), 2 (5.0%) with severe impairment (FEV_1_ = 45.5, 2.1%), and 1 (2.5%) with very severe impairment (FEV_1_ = 25%). Inhaled corticosteroids were used to treat 12.8% of COPD patients. The mean dose of inhaled corticosteroids in COPD patients was 750 (353) mg of fluticasone propionate or its equivalent per day. The most frequent primary neoplasms were adenocarcinomas (39.2%) and squamous cell carcinomas (36.5%). Although all cases underwent operations for suspected neoplasms, 8.1% of the patients had lesions that were either histologically benign or had non-neoplastic pathologies. The most frequent comorbidities, apart from respiratory diseases and neoplasms, were diabetes (16 patients), cardiovascular diseases (10 patients), peptic ulcer disease (7 patients) and liver disease (7 patients). Only three cases had positive cultures either in the bronchoscopic study or during the intervention: two in the COPD group and one in the control group.

**Table 2:** Patient demographics

	**Controls (n = 34)**	**COPD (n = 40)**	P value*
Males (n)	26 (76.5)	37 (92.5)	NS
Age (years)	63.1 (10.7)	66.2 (7.6)	NS
Tobacco history (pack-years)	48.8 (101.3)	51.5 (27.7)	NS
Comorbidities (Charlson index)	2.6 (1.6)	3.9 (1.3)	<0.001
BMI (kg/m^2^)	26.5 (4.5)	25.8 (7.07)	NS
FVC (%)	90.6 (17.1)	93.1 (17.9)	NS
FEV_1_ (%)	86.2 (16.5)	72.2 (16.4)	0.001
FEV_1_/FVC (%)	75.6 (6.3)	61.2 (8.8)	< 0.001

The data are expressed as absolute (relative) frequencies or means (standard deviations), as appropriate. *p values were calculated using Chi-square or Mann–Whitney tests, as appropriate.

BMI: body mass index. FVC: forced vital capacity. FEV_1_: forced expiratory volume in one second. NS: not significant.

### Expression of acute-phase reactants in epithelial cells, macrophages and human lung fibroblasts

**Figure 1:**
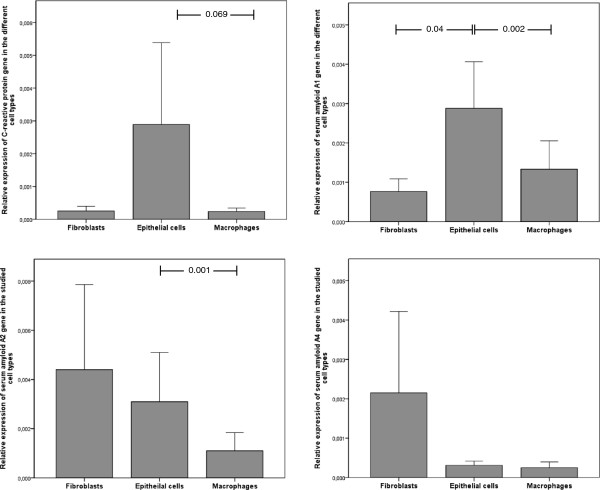
Relative gene expression of a) C-reactive protein, b) serum amyloid A1, c) serum amyloid A2 and d) serum amyloid A4 according to cell type.

All three cell types were capable of synthesizing these biomarkers to some extent. Gene expression was not related to the presence of a neoplasm, the tumor type, TNM staging, inhaled corticosteroid intake, degree of lung function impairment in COPD or presence of a positive microbiological culture. Depending on the cell type studied, we found two different patterns. The CRP and SAA1 genes were more strongly expressed in epithelial cells (Figure 1a and b), whereas the SAA2 and SAA4 genes were more strongly expressed in fibroblasts (Figure 1c and d).

Within one cell type, the gene expression patterns of these biomarkers were correlated. In fibroblasts, there was a strong correlation between the SAA1 and SAA2 genes (r = 0.608, p < 0001). However, there was no evidence of a correlation between SAA1 (r = 0.387, p = 0.001) or SAA2 (r = 0.201, p = 0.086) and CRP. In epithelial cells, the highest correlations were between the different SAA genes (SAA1 vs. SAA2: r = 0.962, p < 0.001), with weaker correlations between the SAA and CRP genes (CRP and SAA1: r = 0.579, p < 0.001; CRP and SAA2: r = 0.452, p < 0.001). However, in macrophages, the correlations were uniformly low across all biomarkers, with r values of approximately 0.4 and p values <0.001 for all comparisons.

### Gene expression of acute-phase reactants in these cell groups between COPD patients and healthy smokers

**Figure 2:**
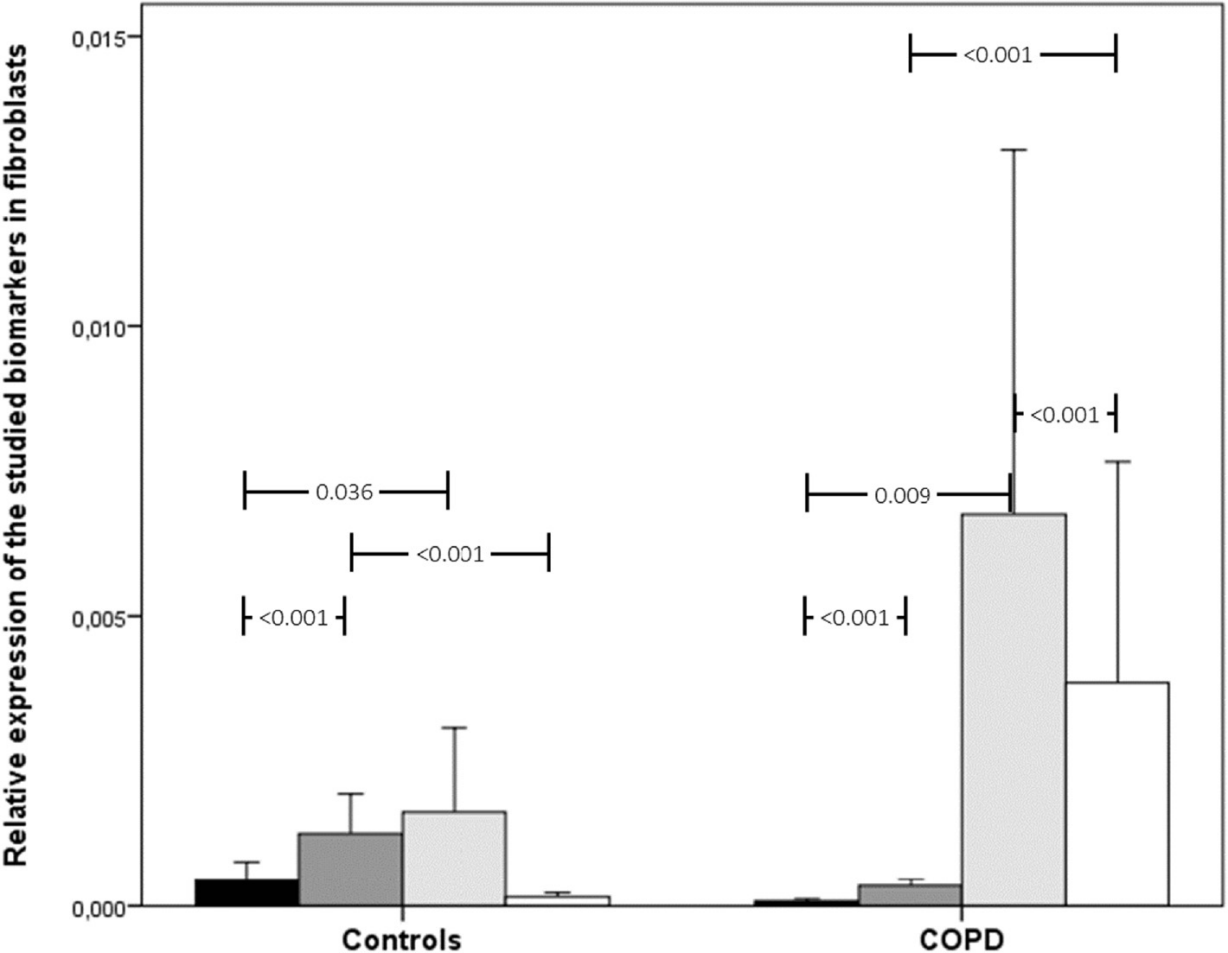
Relative gene expression of acute-phase reactants in fibroblasts in COPD patients and controls.

**Figure 3:**
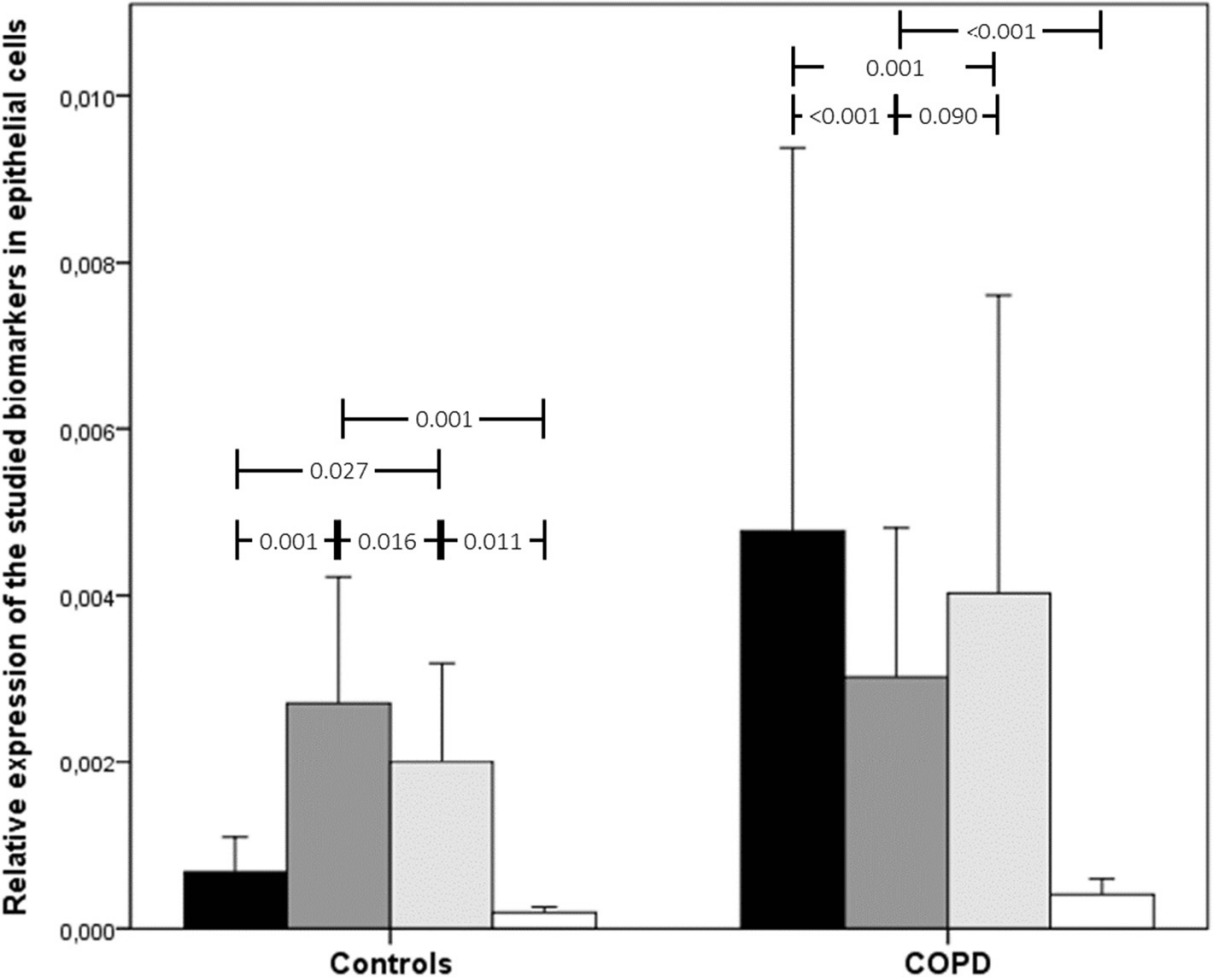
Relative gene expression of acute-phase reactants in epithelial cells in COPD patients and controls.

**Figure 4:**
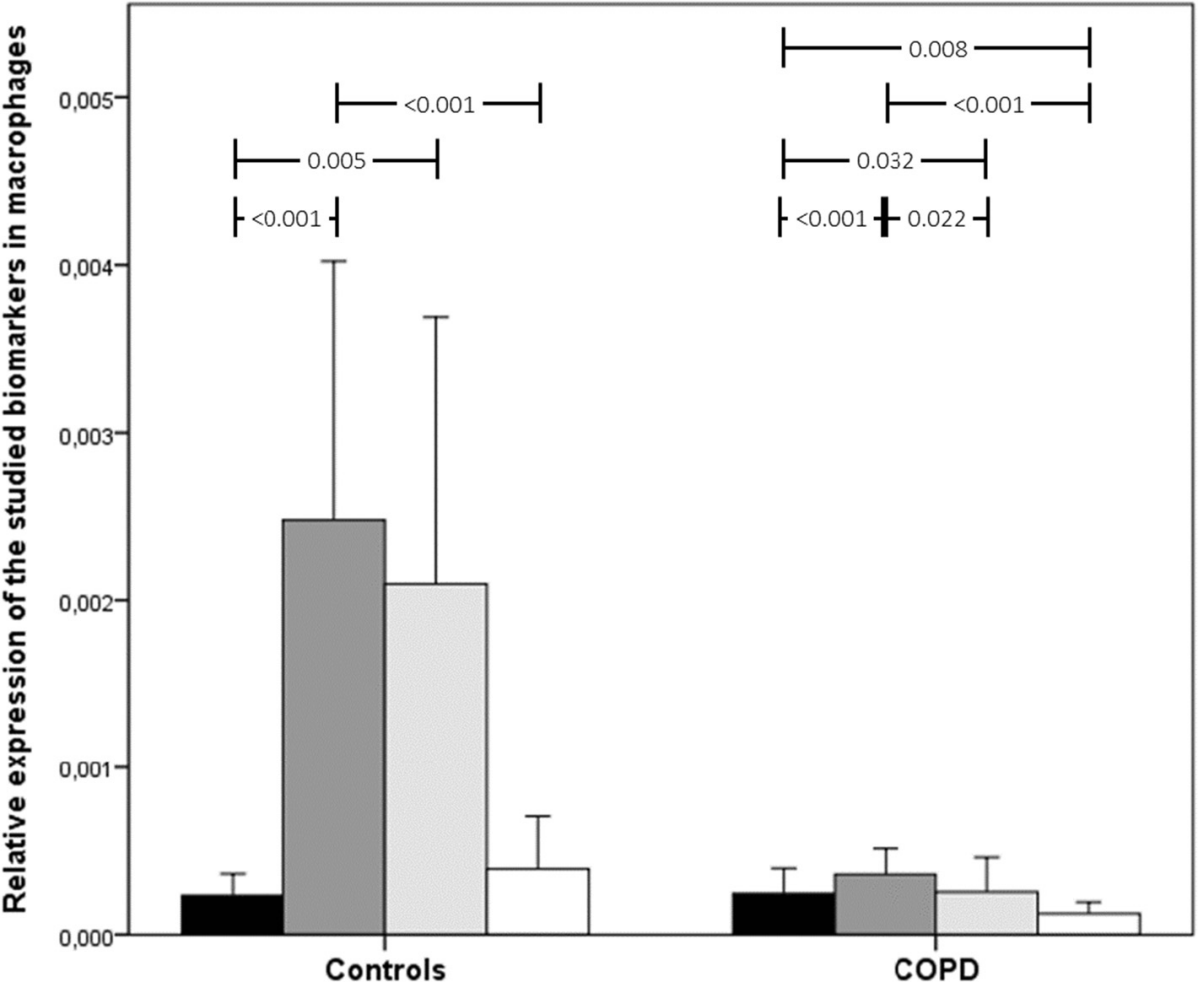
Relative gene expression of acute-phase reactants in macrophages in COPD patients and controls.

When analyzing the gene expression of each biomarker, we observed different patterns depending on the cell type (Figures 2, 3 and 4). In fibroblasts, gene expression of the studied biomarkers was increased in the COPD cases, especially for SAA2 (Figure 2). Thus, gene expression increased for SAA2 and decreased for SAA1 in COPD patients.

In epithelial cells, gene expression was also increased, albeit the difference was less striking (Figure 3). In epithelial cells, there was a striking increase in CRP, which was not observed in fibroblasts or macrophages. In macrophages, however, gene expression was decreased in COPD patients compared to controls, particularly for SAA1 and SAA2, with no change in CRP (Figure 4). The results of multivariate analysis indicated that these results did not appreciably change after adjustment for potential confounders, including body mass index, age, gender, FEV_1_ and the Charlson index.

## Discussion

The present study provides novel information regarding the expression of CRP and SAA in the lung parenchyma. We identified differences in production according to the cell type and the presence or absence of disease. The results of the present study reflect two main findings: firstly, all fibroblasts, epithelial cells and macrophages express CRP and SAA, and secondly, expression of both markers differs according to cell type and the presence of disease.

To correctly interpret the results, several issues should be considered. First, because all the study subjects were admitted for a pulmonary resection (lobectomy or pneumonectomy) for suspected primary lung cancer and therefore met the criteria for surgery, 92.3% of the COPD patients had mildly or moderately impaired lung function. Therefore, this was not representative of all COPD patients because there were few surgical specimens from patients with advanced-stage COPD. Second, the resected neoplasms included several tumor types and several non-neoplastic cases, and it is unclear whether the observed changes were dependent on tumor type. In the present study, histological types were adequately distributed with proportional numbers for each type of neoplasm, and we found no influence of tumor type on the results. Third, although the tissues analyzed were not macroscopically affected by the tumors, it remains unclear whether microscopic influences maybe involved. Data from the diagnostic techniques and current high-resolution images make this possibility very unlikely. However, different genetic predispositions for COPD cases with and without lung cancer have previously been described [19]. Therefore, the results of our project should be strictly applied to patients with COPD with similarly located lung neoplasms. Fourth, because surgery itself can potentially stimulate the studied biomarkers, cases for which the time between the opening of the skin plane and surgical excision exceeded 3 hours were excluded. The described increase in reactants occurs several hours following acute injury and remains elevated for several days [20]. Therefore, three hours was considered a reasonable time for such surgical procedures without complications. Fifth, these results apply to the lung parenchyma. This implies that fibroblasts, macrophages and epithelial cells are obtained, in the best case, in the terminal bronchi and the lung parenchyma. In this regard, the roles of these cells may be more relevant to heavier gauged airways [21]. In contrast, the expected effect of COPD on the parenchyma would be emphysema, which would result from an imbalance between various pathogenetic mechanisms, such as oxidation-antioxidation equilibrium [22], protease-antiprotease equilibrium [23] and potentiation of apoptosis [24]. Consequently, epithelial cells, macrophages and fibroblasts are absent or diminished in number. However, we have been able to demonstrate differential gene expression in the bronchus compared to the parenchyma in COPD patients [14]. Thus, future studies should also include samples from the bronchial airway to complement the present study. Sixth, there are other cell types that maybe potential sources of these biomarkers, including dendritic or endovascular cells. The present study should be expanded to include other cell types to more comprehensively represent the true scenario. Finally, we did not measure serum protein concentrations of these biomarkers to test whether gene expression translates to the serum, which would provide a necessary link to systemic inflammation.

The role of these cell types in the pathogenesis of COPD has previously been studied, and the differential expression patterns in fibroblasts and epithelial cells compared to macrophages merits comment. Our results seem to suggest that epithelial cells and fibroblasts play a major role in inflammatory gene expression in COPD. Bronchial biopsy studies have identified epithelial cells as components of inflammation [25]. Moreover, this relationship is associated with smoking intensity [26]. The predominance of synthesis in epithelial cells of COPD patients could be a consequence of remodeling that occurs in the lung tissue of patients with COPD [27]. In this regard, one potential mechanism contributing to airway fibrosis is the transition of airway epithelial cells to a mesenchymal phenotype expressing myofibroblast characteristics and capable to migrate into the lamina propria. Such a process has been termed epithelial mesenchymal transition (EMT) [28]. When this phenomenon is accompanied by angiogenesis, it can explain the increased risk of malignant transformation predominantly observed in the large airways of COPD patients [29]. The potential role played by EMT in the pathogenesis of COPD is currently a very active area of research [30]. Although this study was not specifically focused on this issue, we believe that it may contribute to the ongoing scientific debate in the field.

Fibroblasts are principally responsible for the production and maintenance of the lung extracellular matrix [31]. These cells are involved in the pathogenesis of COPD, particularly in the small bronchial airway, and their peribronchiolar proliferation has been described as the most limiting factor in the pathogenesis of chronic airflow obstruction [32]. Furthermore, alterations in the functional capacity of these fibroblasts can play a role in the pathogenesis of pulmonary emphysema, which results in a breakdown of the tissue.

Conversely, gene expression in macrophages has the reverse pattern. Macrophages play a very important role in the pathogenesis of COPD [33]. They are central to coordinating immunity against inhaled particles dissolved in smoke due to their role as first-line, innate defenders against chronic noxious stimuli in the airway and lung parenchyma. However, their role in the synthesis of these biomarkers is decreased. One explanation might be that they are dedicated to other inflammatory functions in the context of COPD.

The differences found in our study did not appreciably change after adjusting for potential confounders, including body mass index, age, gender, FEV_1_ and the Charlson index. Although these variables have been associated with the systemic inflammatory load [34,35], few data are available on their relationships with local inflammatory biomarkers in respiratory tissues. In the present study, we failed to identify such an association. However, the current results should be taken with caution because the sample size may be too small for assessing the potential impact of such variables. Future studies may utilize the information provided in this study for determining an adequate sample size to investigate such associations. Moreover, further research is needed to assess the potential relationships of potential confounders with respiratory biomarkers obtained using non-invasive methods [36].

Although in this study we did not specifically measure the systemic inflammatory load of the study participants, one of the most obvious explanations for the presence of systemic inflammation in COPD is that local inflammatory processes occurring in the lung may cause a “spills over” of proinflammatory molecules into the systemic circulation [12]. However, the results of previous studies do not completely support this hypothesis because of the lack of association between airway cytokine concentrations and the corresponding systemic levels [37]. Even though a correlation between serum and tissue expression should be obtained to support the “spill over” hypothesis, mRNA quantification cannot provide reliable information as to whether: a) the mRNA will be translated into a protein, b) a functional protein will be translated and c) if such a protein will be finally released into the circulation [38]. It is also noteworthy that mRNA and protein concentrations do not always correlate [39]. Further functional studies are needed to understand the complex relationships between local and systemic inflammation in COPD patients.

## Conclusions

The present study provides novel data regarding the expression of CRP and SAA genes in the lung parenchyma and indicates which cell types are primarily responsible for gene expression of CRP and SAA. The study also uncovers differences in production according to cell type and the presence or absence of COPD. The results of the present study advance the field’s understanding of COPD pathogenesis and the role of systemic inflammation. Future studies should advance our understanding of gene expression in other cell types and the relationship between gene expression and the final serum concentrations of these proteins.

## Competing interests

The authors declare that they have no competing interests.

The authors did not receive reimbursements, fees, funding, or salary from any organization that may have a financial interest in the publication of this manuscript. Moreover, all authors do not hold any stocks or shares in an organization that may have a financial interest in relation to this manuscript. The authors do not have patent activities related to this manuscript.

## Authors’ contributions

All authors have contributed significantly and have read and approved the manuscript. Moreover, they grant an exclusive licence to the journal in the event of the work being accepted. CC conceived of the study, participated in its design and coordination, recruited patients from the surgical waiting list and draft the manuscript. EA and VSL carried out the molecular studies and participated in the sequence alignment. JLL-C participated in the design of the study, performed the statistical analysis and helped to draft the manuscript. JLL-V and NMM performed the extraction of the anatomical sample during surgery.

## Pre-publication history

The pre-publication history for this paper can be accessed here:

http://www.biomedcentral.com/1471-2466/14/95/prepub
